# Correspondence

**Published:** 1890-03

**Authors:** 


### Correspondence.

NEW YORK, February 12, 1890.

In a recent clinic at the University Medical College, Prof. Alfred L. Loomis spoke against the employment of hydragogue, cathartics and diuretics in the treatment of subacute pleurisy. The method of blood-letting, which was so frequently resorted to in former days, and finally abandoned, on account of its debilitating effects, is, he thinks, no more exhausting than the depletion produced by hydragogue cathartics and diuretics. Though he is disposed to recommend their use in simple dropsy, he has failed to find any evidence pointing to their absorbefacient action as sero-fibrinous fluids, the products of an inflammatory process. He advises food and stimulation for patients suffering from subacute pleurisy, and bases his argument upon the clinical fact that in patients with deposits of lymph in any part of the body absorption takes place much more promptly under the employment of measures calculated to promote the process of nutrition.

In cases in which there is severe pain over the chest, Dr. Loomis advises the use of a blister, but would not recommend its use in debiliated subjects, as it is apt to transform a sero-fibsinous exudation into a sero-purulent one. The accumulation of fluid in the pleural cavity is best treated by early aspiration.

As an illustration of the advanced methods of teaching physiology in this city, it may be mentioned that Prof. John S. Curtis, of the College of Physicians and Surgeons, recently introduced into the lecture room a live Alderney cow, and performed vivisection while the animal was under the influence of ether. He opened the chest in the median line, and exposed to view the heart, thus enabling the students to observe its rhythmical contraction in systole and diastole, an object lesson which afforded much practical instruction on the mechanism of the heart's action.

Prof. Lefferts, in a recent lecture, laid down the following points in the differential diagnosis between hysterical aphonia and aphonia from paralysis of the vocal cords. If, on directing the patient to cough, a phonetic sound is produced, you may be certain that she is hysterical; while in paralysis of the chords, there is no phonetic note whatever.

Prof. Wyeth recently added another case to his long list of successful suprapubic cystotomies. The patient, a man, aged 60, was admitted to the Polyclinic Hospital, with symptoms pointing to stone in the bladder. Four weeks before his admission, the attending physician had attempted to remove the calculus by lithotrity, but only aggravated the existing condition. Prof. Wyeth performed cystotomy, and extracted a piece of rubber tubing about three inches long, which was encrusted with urinary deposits. The patient made a good recovery.

Prof. H. Marion Sims' favorite treatment for vaginitis is to paint the vaginal mucous membrane with a solution of nitrate of silver in the proportions of one drachm to the ounce. This forms a thin film which protects the vaginal wall from all irritation and rapidly relieves the burning and smarting which is so distressing to the patient.

Prof. Thompson, of the New York University Medical College, calls attention to the fact that in persons affected with chronic constipation, the sebaceous glands of the face and nose exude a profuse secretion, and that women, after the menopause, frequently suffer from an obstinate form of acne, which can only be cured by relieving the constipation. Hysteria, sick headache, melancholia and many functional neuroses are caused by the presence of the products of putrefaction in the blood, which are absorbed from the intestine and have a destructive influence on the red blood corpuscles. The disadvantage of the ordinary cathartics in use is that the system soon becomes accustomed to them, and for that reason he recommends the following laxative, which he has employed with marked success:

℞ Sulphur, gr. - - - jii
M Cream of tartar, gr. - - jii.

To be made into a lozenge and taken mornings and nights.

It has heretofore been believed by both physicians and laymen that consanguineous marriages were not calculated to produce good physical and mental qualities in the offspring. Prof. McLane, of this city, questions the correctness of this theory, and on the contrary advocates the intermarriage of blood relations. To quote his exact words, "Consanguinity intensifies family characteristics. If you have a perfectly healthy man, physically, mentally and morally, and were to unite him in marriage with his cousin, who is also perfect, physically, mentally and morally, the progeny from such marriage would be godlike."

Dr. Ferdinand King, formerly of Atlanta, Ga., but now of this city, has had unprecedented success in his management of the International Journal of Surgery, a fact which will no doubt prove of interest to his many friends in the south. The Journal of the Respiratory Organs has passed under the editorial management of Dr. H. Holbrook Curtis, a well-known throat specialist, of this city, who will be assisted by a corps of eminent collaborators in this country and abroad. The Journal is published monthly, and is devoted to diseases of the nose, throat and lungs. P. J. R.

